# Patients’ lived experiences of self-injectable diabetes treatment: A phenomenological study

**DOI:** 10.4102/hsag.v28i0.2359

**Published:** 2023-09-15

**Authors:** Frans N. Ndara, Vistolina Nuuyoma

**Affiliations:** 1 School of Nursing and Public Health, Faculty of Health Sciences and Veterinary Medicine, University of Namibia, Rundu, Namibia

**Keywords:** diabetes, diabetes mellitus, diabetes treatment, patients’ experiences, self-administration, self-care, self-injection, self-management, type 1 diabetes, type 2 diabetes

## Abstract

**Background:**

A diabetes diagnosis has significant implications and affects the individual’s health and social opportunities; it may also carry ethical and cultural consequences, especially when self-injectable treatment is involved. Therefore, it is important to understand lived experiences of patients on self-injectable diabetes treatment to establish initiatives and develop coping mechanisms that may reduce disease morbidity.

**Aim:**

This study explored and described patients’ lived experiences of self-injectable treatment for diabetes mellitus type 1 and 2.

**Setting:**

The study was conducted in the Rundu health district, Kavango east region, Namibia.

**Method:**

A phenomenological qualitative design was used. The sample consisted of 10 purposively selected patients on self-injectable treatment and data were collected through unstructured individual interviews. Data analysis followed an interpretative phenomenological approach. Ethical principles were adhered to, including respect for autonomy, non-maleficence, beneficence, and justice and ethical clearance was obtained.

**Results:**

Self-injectable treatment is cost-effective, promotes self-care, and relieves the burden on nurses and doctors. But it is a lonely journey, causing uncertainty about the future and self-stigmatisation. Moreover, unfamiliarity with injection techniques, challenges in storing medication, and disposing of used needles and other waste were revealed.

**Conclusion:**

Patients on self-injectable diabetes treatment have positive and negative lived experiences. It is recommended that family members provide adequate support and that healthcare workers reinforce education on diabetes for these individuals.

**Contribution:**

The findings can be used to develop patients’ education and training packages, guide the development and implementation of diabetes coping mechanisms, and initiate intersectoral collaboration to assist patients undergoing injectable treatment.

## Introduction

Diabetes mellitus (or diabetes as it is commonly known) is a heterogeneous metabolic disorder characterised by the manifestation of high blood glucose levels as a result of impaired secretion of insulin from the pancreas, defective insulin actions on target cells, or both (Punthakee, Goldenberg & Katz 2018:10). According to the American Diabetes Association (ADA) Professional Practice Committee (2022), there are four general categories of diabetes. Type 1 diabetes is an autoimmune disease attributed to the destruction of B cells in the pancreatic islet, leading to complete insulin deficiency. Type 1 diabetes commonly develops during childhood and adolescence, and is therefore known as juvenile diabetes. Type 2 diabetes is caused by the advanced loss of adequate B-cell secretion of insulin and is frequently associated with insulin resistance. The third type of diabetes typically becomes evident during pregnancy and is diagnosed in the second or third trimester, classified as gestational diabetes mellitus. The fourth category encompasses types of diabetes that may be caused by monogenic diabetes syndromes, exocrine pancreatic diseases such as pancreatitis and cystic fibrosis, and chemical or drug-induced diabetes because of the use of glucocorticoid, side effects from HIV and/or AIDS treatment, and treatment after organ transplantation (ADA 2022:17).

Diabetes is prevalent in all populations globally, including rural parts of low- and middle-income countries (World Health Organization [WHO] 2019:6). The International Diabetes Federation (IDF) (2021:5) estimates that 537 million people at the global level are living with diabetes, and this number is projected to increase to 783 m people by 2045. Moreover, 24m of those diagnosed live in Africa. This reflects an African regional prevalence of 4.5% in 2021. The African region’s projection of people with diabetes may be higher than this, as the continent is reported to have the highest prevalence of undiagnosed diabetes (Kibirige et al. 2019:1). In addition, diabetes is reported to have caused 416 000 deaths in 2021 in Africa (IDF 2021:5). The IDF (2021:5) further reported that countries with a high prevalence of diabetes in Africa include: Tanzania (12.3%), Zambia (11.9%), Comoros (11.7%), South Africa (10.8%), and the Seychelles (8.5%). In Namibia, a survey that tested blood samples from 3278 individuals revealed 5.4% had diabetes, and 6.9% were prediabetic (Adekanmbi et al. 2019:163). The survey was community-based, and there is a likelihood that it excluded people with diabetes who were hospitalised or institutionalised during the data collection period. Community screenings for diabetes are ultimately not encouraged as they may inappropriately test community members at very low risk or test those already diagnosed, and fail to reach the groups most at risk of developing diabetes (Tabaei et al., as cited in ADA 2022:26).

Diabetes is diagnosed when there is hyperglycaemia and characteristic symptoms such as thirst, polyuria, blurring of vision, and weight loss (WHO 2019:5). The diagnosis has significant implications for the individual’s health and may result in potential stigma, affect the person’s life insurance, employment, driving status, social opportunities, and may carry ethical, cultural, and human rights consequences (WHO 2019:6). Treatment is based on the type of diabetes and suspected causes or contributing factors. Typically, treatment is given via oral medication or may be administered as an injection, referred to as self-injectable treatment.

The self-injection treatment approach requires the patient to connect the syringe and needle, measure the required insulin dosage, and execute a sequence of steps to inject themselves (Masuda et al. 2010:485). Alternatively, some patients use a cartridge filled with insulin, to which they connect a micro-needle to inject the required dosage, while others inject themselves with reusable insulin pen devices. Self-efficacy in administering insulin injections is mainly predicted by insulin injection skills, knowledge of diabetes and insulin injection, education level, and illness duration (Huang et al. 2021). Ultimately, self-injectable diabetes treatment often presents various challenges, such as physical fear of injections, erroneous beliefs about insulin, socioeconomic concerns leading to the reuse of needles, and concerns about side effects (Liu et al. 2022:3). Some patients on self-injection treatments revealed a lack of preference regarding medication options, insufficient information regarding insulin, the burden of insulin treatment, treatment concerns, and the desire for social support (Gray et al. 2017:1681). Other patients experienced self-stigma related to their imperfect bodies and were treated as social outcasts, and detested insulin (Nishio & Chujo 2017:169).

Previous studies focused on patients’ self-management knowledge and practice of diabetes mellitus (Mikhael et al. 2019:1); diabetes patients’ self-management challenges and solutions (Masupe et al. 2022:1); self-care experiences of patients receiving insulin treatment (Mekashaw, Demeke & Haile 2022:3); self-care practices of diabetes patients (Tewahido & Berhane 2017:3); patients’ perceptions and preferences of injectable diabetes treatment (Boye et al. 2021:2387; Cosson et al. 2019:251); diabetes patients’ acceptance of injectable treatment (Pantea et al. 2022:5); and self-stigma of patients on type 1 diabetes treatment (Nishio & Chujo 2017). Other studies focused on the impact of patients’ beliefs on insulin acceptance and adherence (Liu et al. 2022), and women’s experiences of using insulin for gestational diabetes (Gray et al. 2017). Generally, there is less focus on the lived experiences of patients on self-injectable diabetes treatment, creating a knowledge gap in this area despite the prevalence of diabetes increasing at a global level (IDF 2021:76); in Namibia, one in five adults is prediabetic (Adekanmbi et al. 2019:162). Moreover, chronic diseases, including diabetes, significantly contribute to deaths in developing countries like Namibia. Therefore, there is a need for empirical research focusing on diabetes mellitus patients’ treatment and their experiences to inform evidence-based practices and care.

The modes of insulin administration have improved to bypass skin barriers in transdermal delivery and enhance the effectiveness of insulin molecules when administered transdermal. Recent advances in transdermal insulin delivery include microneedle-assisted approaches, chemical enhancer-promoted, mechanical forced-triggered and electrically facilitated delivery systems (Zhang et al. 2019:52). With the exception of some microneedle-assisted approaches, other insulin delivery systems listed as advances are not yet available to patients in most middle- and low-income countries, specifically in public or state-managed healthcare services. It is therefore imperative that patients on injectable treatment’s experiences are understood before introducing advanced modes of insulin delivery. To improve the quality of life of patients on self-injectable treatment and promote treatment adherence, it is important to understand their lived experiences. Accordingly, this study was conducted to explore and describe lived experiences of patients on self-injectable treatment for type 1 and 2 diabetes mellitus. The study set out to answer the research question: *What are the lived experiences of patients on self-injectable treatment for type 1 and 2 diabetes mellitus in the Rundu health district?*

## Research methods and design

### Study design

This study applied a qualitative approach in the form of a phenomenological design (Brink, Van der Walt and Van Rensburg 2018:105) that was contextual in nature. Phenomenological studies are entrenched in a philosophical tradition developed by Husserl and Heidegger, and are used to study humans’ lived experiences (Polit & Beck 2017:117). This phenomenological study focused on the lived experiences of individuals on self-injectable treatment for type 1 and 2 diabetes mellitus, with interpretivism as its research paradigm. This design was suitable for the study because it reveals people’s life experiences and what they mean to them.

### Research setting

The study was conducted in the Rundu district, in Kavango east, Namibia. Most of the district receives healthcare services from public health facilities under the Ministry of Health and Social Services, while a small number of people are cared for by private healthcare providers. Private healthcare in the district consists of one medical health centre with approximately 10 consulting rooms operated by medical officers.

### Study population, sample, and sampling strategy

The population was all patients diagnosed with diabetes in the district, while the target population was patients diagnosed with type 1 and 2 diabetes mellitus in the district on self-injectable treatment. This included patients who received type 1 and 2 diabetes mellitus treatment from state and private healthcare facilities. A non-probability purposive sampling strategy was used to select patients diagnosed with type 1 and 2 diabetes mellitus and on self-injectable treatment for at least 1 month. This was done to ensure the study recruited participants with lived experience of self-injectable treatment, and 1 month is adequate to accumulate experience. In addition, prospective participants were only considered if they had resided in the Rundu health district for at least a month while receiving type 1 and 2 diabetes mellitus self-injectable treatment because the study was contextual in nature, and focused on patients from the Rundu district.

Patients diagnosed with diabetes under the age of 18 were excluded from the study. These individuals are considered minor children under the care of parents and guardians. This study focused on adult participants who were legally authorised to make informed decisions regarding their care and treatment. Patients on self-injectable diabetes treatment for less than 1 month were also excluded. The sample size consisted of 10 participants, as determined by data saturation (Hennink, Kaiser & Weber 2019:1483); therefore, the 10 participants sufficiently answered the research question. No patients on diabetes treatment who were approached refused to participate or dropped out of the study.

### Data collection procedures

Data were collected from December 2021 to January 2022 through individual unstructured interviews. After the authors obtained ethical clearance and permission to conduct the study from the University of Namibia and the Ministry of Health and Social Services in Namibia, potential participants were approached at the state and private primary healthcare facilities. The first author (who was the researcher in the field) explained the purpose and objectives of the study, and significant information was included in the participant information sheet. The participants and the first author decided on a time and date for the individual interviews.

The first author conducted all interviews because this researcher could converse in the local languages spoken in the district. Interviews were conducted in English and local languages based on participants’ choices. The interviews conducted in local languages were translated into English with the assistance of a language expert prior to data analysis. An interview guide was used, and communication techniques such as listening, summarising, reflecting, and paraphrasing helped strengthen the dialogue with participants during data collection. The interviews took place at public health facilities in unoccupied consultation rooms, and no other people were present during the interview or listened to the conversations. All interviews were audio recorded with participants’ consent to avoid data loss and assisted the authors in the data analysis process. Additionally, field notes were written during and after the interviews to note the first author’s reflections on participants’ non-verbal responses, body language, and other non-verbal communication cues. The interviews lasted approximately 42 min – 52 min, as determined by participants’ responses.

### Data collection instrument

An unstructured interview guide was used to direct the data collection process. The guide was developed by the authors and consisted of one central question: ‘Tell me about your experience as a patient on self-injectable treatment for diabetes mellitus?’ This was followed by prompts and probes that assisted the authors in understanding participants’ lived experiences. Some probing questions guided participants to talk about their positive and negative experiences of self-injectable treatment, and other probes were based on participants’ responses. The interview guide was pretested with two participants who were not part of the main study. As emphasised by Malmqvist et al. (2019:8), piloting is essential to ensure high research quality when a depth of understanding is required.

### Data analysis

All audio recordings were transcribed verbatim and returned to participants for member checking before data analysis commenced. No software was used during data analysis; the authors analysed data manually using interpretative phenomenological analysis (IPA) (Smith & Osborn 2009:66). The IPA is suitable for use in examining topics that are emotionally laden, multifaceted and vague (Smith & Osborn 2015:41), which was the case in this study. The following analysis steps were followed: transcripts were read a number of times in order for the authors to become familiar with the data and gain new insights. Notes on the authors’ thoughts, similarities, differences and contradictions in participants’ responses were made on page margins. Notes were then transformed into concise phrases intended to capture the essential quality of what was found in the text. The phrases were listed on a sheet of paper, and connections were observed between them. Phrases were interpreted, and similar phrases were grouped together to form themes and subthemes, presented in the form of a coding tree at this stage, and transferred into a table during report writing. While formulating themes and subthemes, the authors also considered the field notes taken during data collection. The two authors met to agree on the final themes and subthemes before writing the final report.

### Measures of trustworthiness

Trustworthiness was ensured using credibility, dependability, confirmability, and transferability (Lincoln & Guba 1985:290–331). Credibility was achieved through prolonged engagement, which means the first author stayed in the field until saturation was reached. This also helped to build rapport and trust with participants, helping the authors to gain an in-depth understanding of their experiences of self-injectable treatment. Furthermore, all interviews were audio recorded to ensure credibility. In addition, peer debriefing was carried out with other researchers not part of the current project. Member checks were performed by taking transcripts and themes generated to the participants in order to confirm interpretations and correct obvious errors. Reflexivity of personal, interpersonal, methodological, and contextual issues was ensured in this research, based on a practical guide to reflexivity in qualitative research (Olmos-Vega et al. 2022:4). This was performed through note-taking in research journals and field notes.

Dependability was achieved by keeping an audit trail of the collected data, transcripts, field notes, methods, and all steps were followed with decisions made during the research process and in writing the final report. In addition, reflexive notes on the authors’ reflections and personal feelings on the topic were made in their research journals. The authors were considered outsiders because they were from an academic institution, were not directly involved in healthcare service provision, and had no relation with participants prior to data collection. In addition, the authors have no experience themselves as patients on self-injectable treatment for diabetes mellitus but possess knowledge of diabetes mellitus and its treatment. However, this did not influence the results and interpretations; through reflexivity, the authors bracketed out their preconceived ideas and beliefs while collecting data, analysing data, and report writing.

Confirmability was achieved through the authors’ reflexivity and triangulation during data analysis, and included extracts of quotes from participants in the presentation of findings. To help other researchers make judgements regarding the findings’ transferability, purposive sampling was employed, data were collected until saturation was reached, and detailed descriptions of the data were provided within the research context.

### Ethical considerations

The study received ethical clearance and permission from the research ethics committee of the School of Nursing and Public Health at the University of Namibia (letter dated 15 November 2021) and the research unit in the Ministry of Health and Social Services (Ref:17/3/3/FNN). Other ethical considerations, according to Dhai and McQuoid-Mason (2011:166–179), included the principles of respect and autonomy, non-maleficence, beneficence, and justice.

## Findings

Ten patients on self-injectable treatment for diabetes mellitus in a selected district participated in the study. The participants’ ages ranged from 23 to 55 years; five were male, and five were female. Seven participants received diabetes mellitus treatment from state healthcare facilities, while three were under private healthcare providers’ care. Table 1 displays all participants’ demographic characteristics.

**TABLE 1:** Participants’ demographics.

Participant	Sex	Age in years	Marital status	Employment status	Year of diagnosis	Private or state patient
1	Male	55	Married	Unemployed	2010	State
2	Male	38	Married	Employed	2016	State
3	Female	23	Single	Unemployed	During childhood	State
4	Female	39	Widow	Employed	2013	Private
5	Female	43	Married	Unemployed	2000	Private
6	Male	28	Single	Employed	2019	State
7	Female	32	Married	Employed	2019	State
8	Male	30	Single	Employed	2002	Private
9	Female	24	Single	Unemployed	2021	State
10	Male	29	Single	Employed	2018	State

Two themes and nine sub-themes were conceptualised from the IPA and are presented in Table 2.

**TABLE 2:** Themes and sub-themes conceptualised from data analysis.

Themes	Sub-themes
1. Positive experiences related to diabetes self-injectable treatment	1.1. Self-injectable treatment is cost-effective 1.2. Self-injectable treatment promotes self-care 1.3. Self-injectable treatment relieves the burden on nurses and doctors
2. Negative experiences related to diabetes self-injectable treatment	2.1. Uncertainty about the future 2.2. Self-stigmatisation 2.3. Self-injectable treatment is experienced as a lonely journey 2.4. Unfamiliarity with injection techniques 2.5. No adequate food or time to prepare a meal before self-injecting 2.6. Challenges related to storing medication, and disposing of used needles and other waste

### Theme 1: Positive experiences related to diabetes self-injectable treatment

#### Sub-theme 1.1: Self-injectable treatment is cost-effective

Participants indicated that because self-injectable treatment occurs at home, in the workplace, or anywhere the patient is, it reduces the cost of taxi fares required for daily injections at healthcare facilities. For patients who received treatment from private care providers, self-injectable treatment reduced costs as they did not have to pay someone to inject them on a daily basis. This treatment approach also reduced patients’ spending on their medical aid and insurance, and avoided them depleting the funds allocated for day-to-day healthcare services. One participant said:

‘As a private patient, injecting myself cut costs of being charged every day. You know, when I have to go to the hospital or pharmacy for injection, I have to pay something so that is it, doing it at home it’s much easier when it comes to costs on my medical aid.’ (Participant 5, 43-year-old female private patient).

Furthermore, participants stated that they adhered to treatment because of its cost-effectiveness:

‘I like the fact that we are allowed to inject self at home, If I have to pay for a cab to go to the hospital just to get this injection, I think I will just give up on this treatment because it is expensive. I’m injecting myself so that cut off the expenses of spending money on a cab going to the hospital and also at the same time, it doesn’t require me paying someone to do it for me, I can afford it. I heard there are some people that employ certain people, let me say health care workers at the end of the month they have to give them something just to care for them for the injection which I do not do, I do it myself, so I don’t have to spend much.’ (Participant 3, 23-year-old female state patient)

#### Sub-theme 1.2: Self-injectable treatment promotes self-care

This study revealed that because patients injected themselves, it promoted self-care and placed them in control of their own health and efforts to regulate blood glucose levels. As patients were actively involved in their treatment, it motivated them to read further about the disease and its treatment, the side effects of treatment, and the correct diet to follow. According to the interview extracts, patients on self-injectable treatment were aware of the side effects and consequences of not injecting themselves, and they knew the right time to inject themselves. Participants explained:

‘Since I started injecting myself, I read a lot about this disease and how to control blood sugar level at home on my own, about side effects I am able to handle them myself. I even feel like I can take care of another diabetic person at home [*smiling*].’ (Participant 5, 43-year-old female state patient)

‘Like me the doctor told me its compulsory to inject myself morning and night, but because I do it myself, I know exactly what hour to inject myself, there will be a sign from my body and if I ignore that, I will have these symptoms, like dizziness, too much hunger or eating too much, so this injection helps me to lower the body sugar without consulting anyone.’ (Participant 6, 28-year-old male state patient)

#### Sub-theme 1.3: Self-injectable treatment relieves the burden on nurses and doctors

Generally, participants in this study observed that nurses and doctors were overworked. Therefore, they were of the opinion that self-injectable treatment for diabetes mellitus may reduce nurses’ and doctors’ workload. Self-injection outpatients did not have to go to the hospital for injections, as required for other diseases such as tuberculosis that require daily injections. This gave nurses an opportunity to focus on seriously ill patients who needed close monitoring and care. Participants mentioned:

‘When you go to the hospital, nurses and doctors are very busy, works is too much for them, hospitals are overcrowded, especially the state hospital. It is good that some of us inject ourselves at home, that way their work is reduced.’ (Participant 7, 32-year-old female state patient)

‘… so it’s also an advantage to the nurses, I cannot be giving unnecessary pressure to the nurses when I can do this myself.’ (Participant 9, 24-year-old female state patient)

### Theme 2: Negative experiences related to diabetes self-injectable treatment

#### Sub-theme 2.1: Uncertainty about the future

This subtheme describes participants’ uncertainty about self-injectable treatment for diabetes mellitus. They mentioned fear about the future, sadness, embarrassment, and irritability. Their fear was also related to injecting themselves and inserting foreign objects into their body; therefore, they questioned using injections for prolonged periods. There were also feelings of uncertainty because participants were unaware of how long they would need to inject themselves and a way forward if their bodies failed to respond to injectable treatment because they were informed this was their last option. Extracts from interview transcripts follow:

‘Injecting self as a diabetic patient it’s like, eish, it’s something that comes with fear, due to the fact that there’s a foreign object, there’s a foreign object that is going under your skin, so to say in the body every day, which is bit fearful unlike tablets which you can swallow every day.’ (Participant 2, 38-year-old male state patient)

‘Even before diagnose with diabetes, I didn’t like injections, I am afraid of injecting myself … what if I don’t do it correctly, what if I harm myself, what if I overdose myself?’ (Participant 5, 43-year-old female private patient)

The fear of needles was elaborated on:

‘… literally I have a fear of needles, I am scared, I have a fear of needles and its very painful, I never liked needles in my whole life, those things really scare me, just by holding it I shake.’ (Participant 7, 32-year-old female state patient)

Another participant mentioned:

‘Living on injection is saddening story, I remember when the doctor explained to me, I was sad, I often ask myself, why me, what is wrong with my body, what went wrong, none of my relative suffer from this disease [*long silence*] … will I ever live a normal life? It’s like I am afraid and sad at the same time.’ (Participant 9, 24-year-old female state patient)

#### Sub-theme 2.2: Self-stigmatisation

Participants cited that self-injectable treatment brought embarrassment and shame into their lives as they did not feel comfortable injecting themselves in the presence of family members, friends, and the public. A participant explained:

‘This injection thing, eish [*shaking head*], remember I do it every day and twice a day, so the injection has changed something about my life, I mean when it comes to my personal luxury life, sometimes I want to go clubbing or spend a night at a friend’s place, I cannot do this because you know when it’s time for injection, I don’t want to inject myself in front of my friends or my girlfriend because this does not look good at all, and they will be wondering why I am angry and irritated, this life …’ (Participant 10, 29-year-old male state patient)

#### Sub-theme 2.3: Self-injectable treatment is experienced as a lonely journey

The interviews with patients on self-injectable treatment for diabetes mellitus indicated they experienced it as a lonely journey. They injected themselves away from others’ presence, and they felt people around them did not care:

‘Every time I inject myself, I have to go in my bedroom, close the door and inject myself. This happen like every evening [*long pause*] … I feel like this is my thing, I have to do it myself, and everyone knows it’s my thing. They don’t ask or interrupt but deep inside me I want to be cared for. I think one day if I forget to inject myself, nobody will even notice.’ (Participant 2, 38-year-old male state patient).

Another mentioned:

‘My people do not even bother to ask if injection is painful, I have to do it, feel it myself, I do not want to say it but I feel nobody care. Even when I go to the hospital, no doctor or nurse will ask how is injecting self-going? They do not even ask if I am coping?’ (Participant 1, 55-year-old male state patient)

In addition, participants expressed that people who are supposed to assist them while they are on self-injectable treatment have limited knowledge and skills about this disease, and are thus unable to support them fully. A participant explained:

‘Coming home, these people I live with are not educated, they don’t know anything about my disease, there’s no one to help me get through the process of injecting myself and all that.’ (Participant 7, 32-year-old female state patient)

#### Sub-theme 2.4: Unfamiliarity with injection techniques

Participants said that despite the number of years they had been receiving treatment for diabetes mellitus, self-injection remained complicated to them because they were not trained healthcare workers. Participants felt that injection required special techniques, such as how to hold a needle, select the right site, and inject. If the incorrect steps are followed, it may lead to pain and discomfort. As a result, some patients may skip doses as they fail to master self-injection techniques. A participant reported:

‘I used to skip some days without injecting myself because I did not know how to do it properly and could not bear the pain, I was doing it wrong, and the pain would make me not want to inject myself.’ (Participant 9, 24-year-old female state patient)

Participants also narrated it was difficult to inject themselves when they were newly diagnosed and when this treatment was introduced to them. It was described as a challenging experience, because participants had little knowledge about diabetes mellitus. It was stated:

‘When I started with injection, it was really a difficult experience for me because I didn’t know how to inject myself but with time yes I improved, because it’s an everyday thing since my life depends on it.’ (Participant 1, 55-year-old male state patient)

Difficulty in injecting themselves was further expressed as follows:

‘It has been a struggle for me, I thought I would never get used to injecting myself, at first I refused to inject myself, but when the nurse explained to me the importance of having to inject myself I then accepted my condition and my situation.’ (Participant 5, 43-year-old female private patient)

Participants stated that it is very painful to self-inject, rather than being injected by someone else. Another factor contributing to pain is limited injection sites, causing the skin to become irritated and painful. They expressed the experience would be better if they were familiar with injection techniques. Moreover, participants indicated that pain at injection sites sometimes interfered with their performance of daily chores:

‘The needle is small but painful, I don’t like that at all, there was a day my abdomen was paining a lot on the surface, and I cannot even work in my garden.’ (Participant 1, 55-year-old male state patient)

Another participant mentioned:

‘The skin on my abdomen and thighs pains all the time, it’s like they do not heal, imagine I have to inject myself morning and evening.’ (Participant 6, 28-year-old male state patient)

Participants indicated that they had tried home remedies to help relieve the pain at injection sites but were unsure of the scientific evidence of their effectiveness:

‘Injection sites can be very painful; I sometimes apply icepacks to relieve pain or rub honey to the area but does not help. I assume those are not correct things to use, you know it’s those things we read on the internet.’ (Participant 8, 30-year-old male private patient)

Participants narrated it was difficult in the beginning, mostly because of their shock at being diagnosed with diabetes mellitus and being told that treatment required them to inject themselves. Consequently, they were dealing with a dual shock:

‘Let me say it’s a difficult thing when I was diagnosed with diabetes; this is a disease that comes with a lot of complications, or problems to say. The worse is that I was put on treatment whereby I have to be injecting myself almost every day of my life and believe me injections are not really good at all.’ (Participant 9, 24-year-old female state patient)

Moreover, participants claimed there was no proper training or guidance on correctly injecting themselves at home, contributing to patients’ unpleasant experiences with this treatment approach. A participant indicated:

‘The injecting part was very difficult for me, it was my first time to hold a needle in my hand, I can say no one guided me on the injecting part, I did not attend any training or any sort of education session, I have to figure it out myself.’ (Participant 1, 55-year-old male state patient)

Unfamiliarity with injection techniques was further expressed as follows:

‘Injecting is too technical for anyone who is not formally trained on how to do it, we really struggle to it correctly.’ (Participant 4, 39-year-old female private patient)

#### Sub-theme 2.5: No adequate food or time to prepare a meal before self-injecting

Some participants found it challenging to prepare meals as they were required to eat before injecting themselves. Injections without eating first led to dizziness, weakness, and a sudden drop in blood glucose levels. Food preparation was described as time-consuming for some, and others reported a lack of access to food to consume before their injection. Participants explained:

‘Another thing is that every time before I inject myself, I have to eat something, I have fainted once because I injected without eating. And again, even if you have not eaten, if you skip the injection, it is a problem again.’ (Participant 3, 23-year-old female state patient)

‘Every time you have to make sure that you eat but sometimes when you don’t have anything to eat it’s a problem.’ (Participant 8, 30-year-old male private patient)

‘I have been on injection for so many years now, I can say it is a lot of work, if I inject without eating, it makes me weak. The difficult is there are moments when I have nothing to eat at home, so with the experience it’s really bad at moments.’ (Participant 3, 23-year-old female state patient)

#### Sub-theme 2.6: Challenges related to storing medication, disposing of used needles, and other wastes

The study revealed that there were various challenges related to storing medication. It is recommended that the insulin used in self-injectable treatment should be kept in a fridge as it is sensitive to warm temperatures. However, not all patients had access to fridges, which is alarming, considering that the temperature in northern Namibia often reaches 40 °C. Some attempted to use cool boxes but could not properly maintain the temperature, as icepacks must be changed regularly. Another challenge related to medication storage was the assumption that keeping insulin in household fridges is dangerous to families with small children as they may confuse it with food or play with it out of curiosity. Extracts from the interview transcripts follow:

‘This diabetic treatment should always be kept in a certain temperature, be it like you store them in a fridge or in a place where there’s much of a cold environment so the main challenge is that if you do not have a fridge, your medication are at risk of getting damaged.’ (Participant 4, 39-year-old female private patient)

‘You know we get a 1-month supply, and with this electricity interruption, it’s difficult to keep medicine cold because electricity can go even for 2 full days, I get worried that my injection might spoil.’ (Participant 5, 43-year-old female private patient)

‘I do not have my own fridge for medication only, I am always afraid that the small ones might play with my insulin container in the fridge, you know children are curious, or some might think its food.’ (Participant 1, 55-year-old male state patient)

Participants expressed concerns about storing and disposing of used needles and other waste from injection procedures. Some stored used needles in empty cold-drink bottles and took these to the clinics for disposal, while others disposed of the items with other household waste. Storing and disposing of needles and other waste was expressed as a major challenge among participants, and they raised concerns about the danger used needles posed to other family members and the environment. This was mentioned:

‘It’s difficult to throw away used needles and those cotton things I sometimes use to clean myself with. You know I cannot keep them at home in a container because what if children play with them so I have to throw them away regularly but eish, it’s a burden.’ (Participant 7, 32-year-old female state patient)

‘I am a bit worried about where to keep the used things you know, what if a child prick self with it or maybe a child inject self.’ (Participant 1, 55-year-old male state patient)

## Discussion

The study explored the lived experiences of patients on self-injectable treatment for type 1 and 2 diabetes mellitus. Participants were aged 23–55 years, which corresponds with the global age of people commonly diagnosed with diabetes (which is 20–79 years), and the age group mostly living with diabetes in Namibia (Ogle et al. 2022:12). The findings revealed that participants had both positive and negative experiences with diabetes self-injectable treatment. Under the positive experiences, self-injectable treatment was considered beneficial to patients and healthcare providers because it is cost-effective, promotes self-care, and relieves the burden on nurses and doctors. A diabetes diagnosis leads to a financial burden associated with dietary changes, the cost of treatment, and transport fees needed to acquire treatment. However, in this study, self-injectable treatment was considered a cost-effective option performed by the patients themselves. There was no need to hire healthcare professionals or spend money on transport to reach healthcare facilities. Another study focused on the cost-effectiveness of self-injectable contraceptives, which was found to have economic benefits for patients because of reduced trips to hospitals (Di Giorgio et al. 2018), which was also the case in this study.

Self-care among patients with diabetes involves self-monitoring blood glucose levels, appropriate dietary practices, regular exercise, adherence to medication, and regular foot care (Tewahido & Berhane 2017:1). Participants in this study indicated that self-injectable treatment is a facilitator of self-care because they are required to eat before injecting themselves, they are aware of the correct diet to follow, and monitor their blood glucose levels regularly because they know the symptoms of hypoglycaemia. Participants did not refer to the use of glucometers to monitor their hypoglycaemia and hyperglycaemia symptoms before self-injecting. Mekashaw et al. (2022:5) similarly reported that patients with diabetes self-monitor blood glucose by being conscious of the symptoms of hypoglycaemia and hyperglycaemia without using a glucometer. These symptoms were used as indicators to remind patients of their medication time, which was also alluded to in this study.

Another positive experience of diabetes self-injectable treatment reported by participants is that it relieves the burden of care placed on nurses and doctors. Many public healthcare facilities experience a shortage of nurses and doctors, coupled with high patient loads. Most participants in this study obtained their diabetes treatment from public healthcare facilities, where they witnessed the demands placed on healthcare providers. Considering that diabetes is common among adults residing in low- and middle-income countries (Ogle et al. 2022:^2^), self-injecting at home prevents patients from queueing at healthcare facilities for treatment, allowing healthcare professionals to focus on other tasks. This finding concurs with Zimmer et al. (2015:278), who reported that self-injection treatments could alleviate pressure on health services by reducing hospitalisations, clinic appointments, and visits to healthcare professionals for routine administration of injections.

Participants’ uncertainty about the future related to their fear and unpredictability of disease progress, sadness, embarrassment, and irritability. Participants in this study were still in their reproductive years, and these feelings of uncertainty are unsurprising after a diagnosis of diabetes. Pikkemaat, Boström and Strandberg (2019:5) documented personal feelings of guilt, denial, shame, and disappointment among this population. While this study reported on participants’ fear of inserting foreign objects into their bodies, a previously study by Wibisono et al. (2017:93) also documented that fear-related factors included past experiences of injections, insufficient knowledge of insulin and its side effects, and misconceptions. The study’s findings on the pain associated with self-injectable treatment concur with Liu et al. (2022:3), who reported that patients experienced pain during self-injection, which leads to needle phobia. According to the participants’ demographic characteristics, most were diagnosed with diabetes within the past 5 years and likely had not mastered the self-injection technique.

Moreover, participants in this study experienced self-injectable treatment as a lonely journey. This implies that patients felt inadequately supported and therefore may lack motivation to continue with treatment. This finding is supported by Pamungkas, Chamroonsawasdi and Vatanasomboon (2020:257), who revealed that lack of social support, role models and family conflict are hindrances to self-management among patients on diabetes treatment, including self-injectable treatment. In some cases, patients felt they had to learn everything about how to live with diabetes themselves, and health professionals did not explain or discuss how to deal with the illness on a daily basis (Van Smoorenburg et al. 2019:4). Therefore, they felt unsupported. Opposite findings were documented by Mphasha, Mothiba and Skaal (2022:6), who revealed patients on diabetes treatment received support from family members in terms of food, exercise, and collection of medication. Family members’ support for patients living with diabetes is central to coping with the disease, better outcomes, general well-being, and preventing complications. Most participants in this study were single, and their feelings of being unsupported and lonely may be related to their marital status.

Challenges with self-injectable treatment included unfamiliarity with injection techniques, reusing pen needles, only using one injection site, not maintaining insulin pressure on the injection button for at least 10 s before withdrawing the needle and applying excess pressure to the skin when injecting (Bari et al. 2020:2598). Netere et al. (2020:6) discovered that patients skipped critical steps or performed them incorrectly when shaking cloudy insulin solution, pinching the skin, injecting at a 45-degree angle, and withdrawing insulin from the vial. This study reported injection technique concerns in how to hold a needle, select the right site, and inject. Challenges with injecting the self were mostly experienced by patients newly diagnosed with diabetes.

According to Tezera et al. (2022:5), the majority of patients on diabetes treatment were found to be food insecure. Similarly, some participants in this study revealed they did not have access to adequate food and consequently missed injection doses to avoid the related complications. In addition, those with sufficient food at home indicated it was time-consuming to prepare a meal before self-injecting. Nutrition is fundamental to the effectiveness of insulin therapy, and self-injecting without eating for long periods leads to complications. Thus, most clinical guidelines recommend that patients on insulin injection administer it not more than 30 min before meals, although insulin post-meals is also acceptable when needed (Schaper et al. 2017:1321). With that understanding, participants in this study risk developing complications because of self-injecting without eating, skipping, or delaying their injection doses.

Challenges related to storing medication, disposing of used needles and other waste were reported in this study. Patients without access to refrigerators in their homes expressed concerns about the insulin solution spoiling. In a study conducted in north-east Ethiopia, the most popular method to store insulin was in a container filled with moistened sand (Bayked, Kahissay & Demeke 2022:6). Moreover, the current findings indicated some patients stored insulin in cool boxes filled with ice cubes and ice packs. This could be risky as melting ice cubes may cause insulin’s immersion; pierced insulin vials carry a high risk of contamination when immersed in water, leading to a loss of potency and the possibility of causing injection abscesses (Bahendeka et al. 2019:346). Patients without refrigerators, because of economic disadvantages, evidently use alternative cooling strategies (Bayked et al. 2022:6), but a lack of adequate refrigeration was viewed as an obstacle to effective insulin storage, which was also the case in this study. Considering that the study was conducted in an area with no constant electricity supply, the reported challenges in storing self-injection medication are unsurprising.

The WHO recommends that used sharp instruments should be placed in puncture-resistant containers made either of a bottle, hard box, or plastic. They should never be disposed of by flushing down the toilet, in recycling bins, public trash cans or household bins, as those put community and household members at risk of being harmed and transmitting diseases (WHO 2016). Regarding the storage and disposal of used needles and other waste, the study’s findings revealed that used needles were stored in empty cold-drink bottles and taken to the clinic for disposal. Some patients disposed of them with other household waste. However, this is a major concern because of the risk of infections and needle prick accidents among family members, especially children. A study conducted in Senegal revealed people who practice self-injection store used devices in empty household containers with a lid until they can be safely discarded. Some patients return used injection devices to the clinic for disposal, while others dispose of used injection devices in pit latrines (Cover et al. 2017:208).

This study included participants who receive diabetes treatment from state clinics and those under the care of private healthcare providers. Therefore, broader perspectives and understanding of their lived experiences were presented through the lens of state and public healthcare services. As a limitation of the study, data collection revived negative experiences among some participants, and they were observed to be emotional. As a result, the first author was patient during data collection and allowed participants to display their emotions. An option for referrals to a state social worker for psychological support was also made available to all participants.

## Study implications

This study’s findings may be used to develop education and training strategies for diabetes patients on proper injection techniques, insulin storage, and the storage and disposal of used needles and devices. Because self-injectable treatment was experienced as a lonely journey and evoked negative personal feelings, the findings may inform the development and implementation of coping mechanisms to reduce disease morbidity and improve patients’ mental health. Thus, these findings have implications for the development of patient education on coping mechanisms, support mechanisms, and referral systems, and may lead to improved food security for patients on diabetes self-injectable treatment.

## Conclusion

This phenomenological study described the lived experiences of patients on self-injectable treatment for diabetes. Overall, patients mentioned that self-injectable treatment was beneficial to them and healthcare service providers, although this approach had some challenges. As the study was conducted in the context of a developing country, food security for patients on diabetes treatment may be improved by encouraging backyard gardening, consuming locally available foods, and setting up small income-generating projects. For patients with access to food but no time to prepare meals before injecting themselves it is recommended that healthcare workers educate them on the use of personal daily schedules. It is also recommended that communities form support groups for patients on diabetes self-injectable treatment, emphasising family involvement in treatment, and the establishment of proper referral systems for psychosocial support when needed.
